# Supplementary material for: Single-Cell Transcriptome Sequence Profiling on the Morphogenesis of Secondary Hair Follicles in Ordos Fine-Wool Sheep
Source: Int J Mol Sci. 2024 Jan 2;25(1):584. doi: 10.3390/ijms25010584 (PMC10779399; doi:10.3390/ijms25010584)
Supplement: Supplementary file 1 [file ijms-25-00584-s001.zip › ijms-2756429-supplementary.pdf]

Table S1 Official Full Name

Gene	Official Full Name
COL1A2	collagen type I alpha 2 chain
COL1A1	collagen type I alpha 1 chain
LUM	lumican
OGN	osteoglycin
DCN	decorin
APOD	apolipoprotein D
CENPF	centromere protein F
TOP2A	DNA topoisomerase II alpha
UBE2C	ubiquitin conjugating enzyme E2 C
TPX2	TPX2 microtubule nucleation factor
LRIG1	leucine rich repeats and immunoglobulin like domains 1
SOX9	SRY-box transcription factor 9
KRT15	keratin 15
KRT5	keratin 5
KRT17	keratin 17
KRT1	keratin 1
KRTDAP	keratinocyte differentiation associated protein
KRT10	keratin 10
SBSN	suprabasin
MGST1	microsomal glutathione S-transferase 1
TYRP1	tyrosinase related protein 1
MLANA	melan-A
DCT	dopachrome tautomerase
TPM2	tropomyosin 2
ACTA2	actin alpha 2
CTSS	cathepsin S
RGS1	regulator of G protein signaling 1
FCER1A	Fc epsilon receptor Ia
LCP1	lymphocyte cytosolic protein 1
RGS10	regulator of G protein signaling 10
LTC4S	leukotriene C4 synthase
PECAM1	platelet and endothelial cell adhesion molecule 1
KDR	kinase insert domain receptor

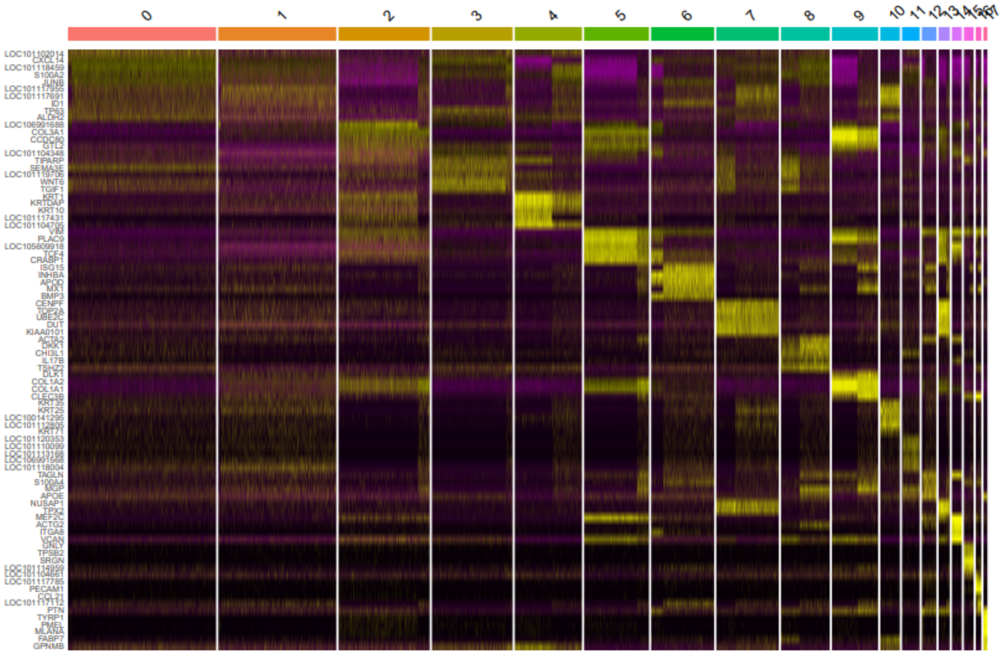

Figure S1 Heat map showing the top 10 differential genes in the subpopulation

Table S2 Antibody information

antibody	catalog	source
Cytokeratin 14 Monoclonal Antibody(LL002)	MA5-11599	Thermo Fisher Scientific
Cytokeratin 15 Monoclonal Antibody(LHK15)	MA5-11344	Thermo Fisher Scientific
Phospho-TLR3(Tyr759) Polyclonal Antibody	PA5-118722	Thermo Fisher Scientific
Sheep anti-Rat IgG(H+L) Secondary Antibody	PA1-28642	Thermo Fisher Scientific
Goat anti-Mouse IgG(H+L) Cross-Adsorbed Secondary Antibody	A-11001	Thermo Fisher Scientific

Table S3 List of principal reagents and instruments

reagentsand and instruments	producer
4% polyformaldehyde	Biyuntian Biotechnology Co., LTD
absolute ethyl alcohol	Nanjing Shengqinghe Chemical Co., LTD
xylene	Merck Life Sciences LTD
Hematoxylin dye	Merck Life Sciences LTD
Eosin dye	Biyuntian Biotechnology Co., LTD
biological microscope（XSP-13CA）	Shanghai optical instrument Factory
paraffin slicing machine（LEICAEG2265）	Leica Microsystems (Shanghai) Trading Co., LTD
Slicing blade（E0997)	Leica Microsystems (Shanghai) Trading Co., LTD
Tissue encapsulation box	Thermo Fisher Scientific

Table S4 Summary of dataset quality

sample	E93	E110
Estimated Number of Cells	12,929	18,892
Valid Barcodes	96.6%	97.3%
Mean Reads per Cell	34,629	26,828
Median Genes per Cell	1,946	1,589
Total Genes Detected	19,997	20,174
Reads Mapped to Genome	88.3%	93.4%
